# Effect of Yangyinqingfei decoction on radiation-induced lung injury via downregulation of MMP12 and TIMP-1 expression

**DOI:** 10.3892/etm.2014.1686

**Published:** 2014-04-24

**Authors:** HONGXIA LI, HONGYING WU, YUE GAO, SHAOHUA CAI

**Affiliations:** 1 Department of Respiratory Medicine, South Building, Special Inpatient Unit, Chinese PLA General Hospital, Beijing 100853, P.R. China; 2 Department of Respiratory Medicine, Special Inpatient Unit, Chinese PLA General Hospital, Beijing 100853, P.R. China; 3 Institute of Radiation Medicine, Academy of Military Medical Sciences, Beijing 100039, P.R. China

**Keywords:** lung injury, matrix metalloproteinases, tissue inhibitors of metalloproteinases, Traditional Chinese Medicine

## Abstract

The aim of this study was to evaluate the effect and underlying mechanism of Yangyinqingfei decoction on radiation-induced lung injury in rats. Wistar rats (n=75) were randomly divided into five experimental groups (A-E). Rats in two of the groups were administered saline solution, whereas rats in the remaining three groups were administered different doses of Yangyinqingfei decoction. After one week, the rats were irradiated with a single dose of 25 Gy to their right hemi-thoraxes by a ^60^Co γ-ray, with the exception of the control group, which underwent sham irradiation. The effect of Yangyinqingfei decoction was assessed one, two and four weeks post-irradiation according to the pathological changes and the right lung index (wet weight of right lung/body weight ×100%). Expression levels of matrix metalloproteinase-12 (MMP-12) and tissue inhibitors of metalloproteinases-1 (TIMP-1) in lung tissue were determined using the reverse transcription-polymerase chain reaction and western blot analysis. Pretreatment with Yangyinqingfei resulted in a significant dose-dependent resistance to radiation-induced body weight loss. The expression of MMP-12 and TIMP-1 increased following irradiation. However, the levels of MMP-12 and TIMP-1 in groups receiving Yangyinqingfei were lower four weeks after irradiation compared with those in rats administered saline. Cumulatively, these results suggest that Yangyinqingfei has a protective effect on radiation-induced lung injury in rats, possibly by downregulating MMP-12 and TIMP-1 expression.

## Introduction

One of the most common complications following radiotherapy for the treatment of breast tumors is radiation-induced lung injury. Two distinct clinical stages are recognized in radiation-induced lung disease: An early, transient stage characterized by radiation pneumonitis and a later stage characterized by chronic radiation fibrosis, impacting on local tumor control rate, prognosis and quality of life following radiotherapy (1).

The mechanism of radiation-induced lung injury has not yet been fully elucidated. It has been suggested that the most important pathological basis of radiation-induced lung injury is a metabolic imbalance in the extracellular matrix (ECM) (2). Matrix metalloproteinases (MMPs) and tissue inhibitors of metalloproteinases (TIMPs) are important in ECM degradation and remodeling (3,4). Numerous studies have found that MMPs have a key role in acute respiratory distress syndrome, chronic obstructive pulmonary disease, idiopathic pulmonary fibrosis (IPF) and lung disease caused by fibrosis (5–8). Yang *et al* (9) revealed that basement membrane rupture in radiation-induced lung injury is closely associated with high expression of MMP-2 and MMP-9. Another member of the MMP family, MMP-12, is produced primarily by macrophages and is capable of degrading a broad spectrum of substrates. MMP-12 is associated with a variety of diseases, including atherosclerosis and lung cancer (10,11). Animal studies have shown that MMP-12 is involved in the induction of the inflammatory response, degradation of the ECM, airway remodeling and the regulation of other metalloproteinases (MMP-2 and MMP-9) and cytokines (12). Matute-Bello *et al* (13) demonstrated that increased expression of MMP-12 causes a progressive pulmonary fibrosis associated with increased fibrosis gene activation during the early stages of lung injury in mice, whilst MMP-12 gene-knockout mice do not develop pulmonary fibrosis. Another study found that MMP-12 protein expression was significantly increased in the lung tissue of rats following right chest irradiation (14). TIMPs, which act as major regulators of MMPs, are capable of inhibiting matrix degradation and maintaining homeostasis in the ECM. Animal studies have reported that TIMP-1 and TIMP-2 are highly expressed in hepatic fibrosis (15) and hyperoxia-induced acute lung injury (16). Recently, a recovery of the imbalance in MMP/TIMP levels was observed in a rat model of lung fibrosis following treatment with Cordyceps in preventive and therapeutic regimens (17). Thus, MMP-12 and TIMP-1 may have an important role in the incidence of radiation-induced lung injury.

Traditional Chinese Medicine, or herbal medicine, is an important approach in the treatment of lung injury. An effective treatment series, known in Chinese as Yangyinqingfei, has been developed by physicians to decrease inflammatory mediators of the lung. Yangyinqingfei decoction is believed to expel wind, eliminate dampness and promote blood circulation to ameliorate pain, invigorate the spleen and regulate qi (18). Yangyinqingfei decoction has traditionally been used for the treatment of diphtheria (19). There are few reports on this herbal remedy, particularly with regard to the mechanism underlying the treatment of radiation-induced lung injury. In this study, the therapeutic effects of Yangyinqingfei decoction were evaluated in a rat model of radiation-induced lung injury, and the potential mechanism underlying the effect was investigated.

## Materials and methods

### Yangyinqingfei decoction

Yangyinqingfei decoction was purchased from the Traditional Chinese Medicines Pharmacy of the People’s Liberation Army General Hospital (Beijing, China). The Yangyinqingfei decoction prescription consisted of the following five Chinese herbs: Sheng Di Huang (15 g), Xuan Shen (15g), Verbena (15 g), Forsythia (10 g), and Gan Cao (6 g). The quantity of each herb in Yangyinqingfei decoction was determined using information from the Pharmacopoeia Commission of the People’s Republic of China (20). Briefly, each herb was decocted by simmering in water for 30 min, prior to being filtered through filter paper and then concentrated into decoctions of 0.2, 0.6 and 1.8 g/ml. The extracts were stored at 4°C until use.

### Animal conditions and treatments

Seventy-five male Wistar rats, aged 6–8 weeks and with body weights of 200±20 g, were obtained from the Experimental Animal Center of the Academy of Military Medical Sciences (Beijing, China). All animal experiments were approved by the Veterinary Institute of the Academy of Military Medical Sciences Animal Ethics Committee. The rats were housed at 23±2°C and 55±5% humidity with a standard 12-h light/dark cycle. The rats had free access to water and were fed a normal diet. After three days of adaptation, the rats were randomly divided into five groups (n=15/group): Control rats with sham irradiation and without drug administration (group A); irradiated rats without drug administration (group B/model group); irradiated rats with low-dose drug administration (group C); irradiated rats with intermediate-dose drug administration (group D) and irradiated rats with high-dose drug administration (group E). All the rats were irradiated with a single dose of 25 Gy to their right hemi-thoraxes by a ^60^Co γ-ray, with the exception of rats in group A, which underwent sham irradiation. One week prior to irradiation, the rats in group A and B were administered saline, while rats in groups C, D and E were simultaneously administered Yangyinqingfei decoction at a dosage of 2, 6 and 18 g/kg body weight/day, respectively. All drugs were administered by intragastric administration once a day until the rats were sacrificed.

### Histopathological examination

At one, two and four weeks post-irradiation, five animals in each group were sacrificed and the lung tissues were harvested. The right lung tissues were paraffin-embedded and sectioned at a thickness of 5 μm prior to being stained with hematoxylin and eosin (H&E) and observed under a light microscope [Olympus (BH-2), Nagano, Japan].

### Western blot analysis

To assess protein levels of MMP-12 and TIMP-1 in lung tissues, western blot analysis was performed. Lung tissues were adequately homogenized with non-denaturing lysis buffer and centrifuged at 12,000 × g for 15 min at 4°C. The protein concentration of the supernatant was determined, prior to denaturation of the protein using a protein loading buffer. A total of 30 μg protein was then loaded onto a 12% SDS polyacrylamide gel, blotted onto a polyvinylidene difluoride membrane and blocked for 1 h with 5% skimmed milk in Tris-buffered saline with 0.05% Tween 20. Membranes were incubated with primary antibodies against MMP-12, TIMP-1 and β-actin (1:1,000; Takara Bio, Inc., Shiga, Japan) overnight at 4°C followed by a 2-h incubation with a horseradish peroxidase-conjugated secondary antibody (1:3,000; Santa Cruz Biotechnology, Inc., Santa Cruz, CA, USA). The membranes were washed with TBS-T buffer three times and then visualized using enhanced chemiluminescence (ECL; Amersham, Piscataway, NJ, USA) detection.

### RNA extraction and reverse transcription-polymerase chain reaction (RT-PCR) analysis

Total RNA was extracted from lung tissues with TRIzol reagent (Invitrogen Life Technologies, Karlsruhe, Germany). Total RNA (1 μg) was reverse-transcribed to cDNA, which was then used to determine the levels of MMP-12 and TIMP-1 mRNA using PCR with Taq DNA polymerase (Fermentas, Pittsburgh, PA, USA). PCR was performed under the following conditions: Initial denaturing at 94°C for 30 sec, annealing at 58°C for 30 sec and extension at 72°C for 1 min, followed by 30 cycles of amplification for MMP-12 and TIMP-1 and 35 cycles for β-actin. The primer sequences for MMP-12, TIMP-1 and β-actin were designed with Primer Premier 5.0 software (Premier Biosoft, Palo Alto, CA, USA) and were as follows: MMP-12 forward 5′-AGGTCAAGATGGATGAAGCGG-3′, reverse 5′-GAAGTAATGTTGGTGGCTGGACTC-3′; TIMP-1 forward 5′-ACAGCTTTCTGCAACTCG-3′, reverse 5′-CTATAGGTCTTTACGAAGGCC-3′; β-actin forward 5′-TGGCCTCACTGTCCACCTTC-3′, reverse 5′-CGAATGGCTGACCATTCAGA -3′. All processes were performed according to the manufacturer’s instructions. Samples were then analyzed using gel electrophoresis (2% agarose), DNA bands were examined and the level of DNA was measured semiquantitatively using a Gel Documentation System (Bio-Rad Model Gel Doc 2000; Bio-Rad, Hercules, CA, USA).

### Statistical analysis

Data are presented as mean ± standard deviation. Statistical evaluation of the results was performed using analysis of variance with a Tukey post hoc test. P<0.05 was considered to indicate a statistically significant difference.

## Results

### Effect of Yangyinqingfei decoction on clinical signs in rats with radiation-induced lung injury

Throughout the experiment, rats in the control group (group A) were active, exhibited good spirits, had normal food intake and showed no obvious abnormalities in the stools. However, rats in group B presented with anepithymia, inactivity, ruffled fur, redness of the nose and dry stools, as well as skin erosion and ulcers. In comparison with group B, rats in groups C-E exhibited alleviated symptoms, and had no skin erosion or ulcers. Among groups C-E, rats in group D exhibited the mildest symptoms, and rats in group E had diarrhea.

The increase of body weight of rats in groups B-E was markedly slower than that of rats in group A. One week after irradiation, the body weights of rats in group D were significantly increased compared with the model group (group B), while no significant differences were observed among the body weights of rats in groups B, C and E. Two weeks after radiation, the body weights of rats in group C and D were significantly higher than those of rats in group B. However, no significant difference was identified between the body weights of rats in group E and those of rats in group B (Table I). These results suggest that Yangyinqingfei decoction may prevent weight loss in irradiated rats.

### Histopathological effects of Yangyinqingfei decoction on rats with radiation-induced lung injury

Gross observations revealed that the right lungs of group A rats were smooth, pink, shiny and elastic throughout the duration of the study. In group B, the surfaces of the right lungs exhibited congestion without other obvious changes in morphology at one week post-irradiation, while at two weeks post-irradiation more serious swelling was apparent in the right lungs than in the left lungs. Four weeks after irradiation, the surfaces of the right lungs of group B rats exhibited petechiae, visible white nodule-like structures and pleural effusion. However, fewer lesions of the right lungs were observed in groups C-E compared with group B (data not shown).

**Figure 1:**
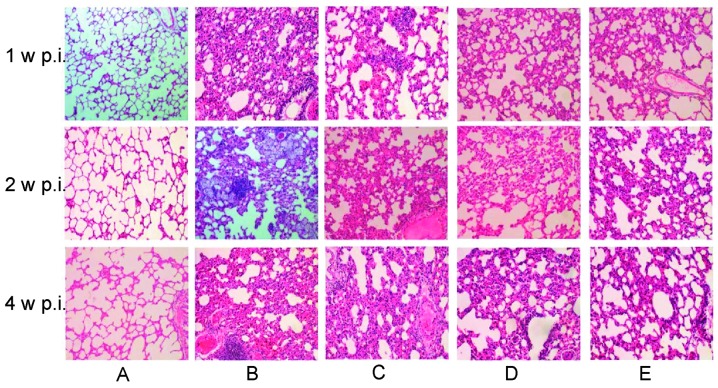
Histopathological changes of lungs following irradiation with or without Yangyinqingfei decoction treatment. (A) Control group; (B) model group (irradiated rats without drug administration); (C) low-dose treatment group (2 g/kg/day); (D) intermediate-dose treatment group (6 g/kg/day); (E) high-dose treatment group (18 g/kg/day). Rats in group A received sham irradiation; others were irradiated with a single dose of 25 Gy to their right hemi-thoraxes by a ^60^Co γ-ray. Rats were sacrificed one, two or four weeks after irradiation and the lung sections showed different degrees of congestion and collapsed alveoli following ^60^Co γ-ray irradiation with or without Yangyinqingfei decoction administration. Hematoxylin and eosin staining; magnification, ×200. p.i., post-irradiation.

Photomicrographs of lung specimens stained with H&E are shown in Fig. 1. Photomicrographs of Group A lungs show normal alveolar structure, comprising of a thin alveolar wall without exudate or effusion. In group B rats, at one week post-irradiation the right lung showed mild edema, capillary congestion, a small amount of exudate and inflammatory infiltration, while at two weeks post-irradiation, part of the alveolar structure had disappeared. In addition, significantly thicker alveolar septa and more notable inflammatory invasion were observed. Four weeks after irradiation, severe perivascular inflammatory infiltration, interstitial congestion, macrophage and lymphocyte accumulation and alveolar wall thickening were observed. Part of the alveolar space had also collapsed or disappeared, while part of the alveolar space had expanded compensatorily. The formation of emphysema, visible fiber cell hyperplasia and focal fibrosis were also observed in group B. Comparatively, the pathological changes in the lungs of group C-E rats were milder, among which the mildest changes were observed in group E. These findings indicate that Yangyinqingfei decoction may alleviate lung injury induced by radiation.

### Effects of Yangyinqingfei decoction on expression of MMP-12 and TIMP-1

**Figure 2:**
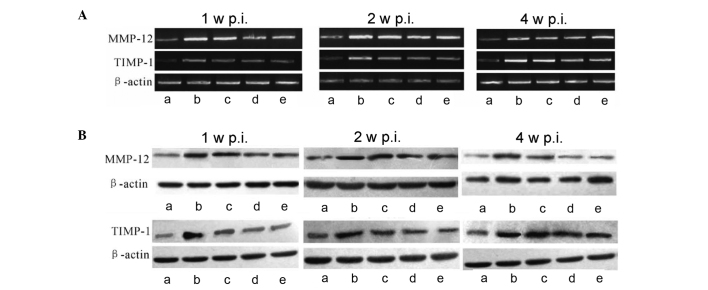
Effect of Yangyinqingfei decoction on MMP-12 and TIMP-1 expression. At different time-points following irradiation, total RNA was extracted from lung tissues and reverse transcription-polymerase chain reaction and western blot analysis were performed. (A) mRNA expression of MMP-12 and TIMP-1 one, two and four weeks after irradiation; (B) protein expression of MMP-12 and TIMP-1 one, two and four weeks after irradiation. β-actin was used as an internal control. a, Control group; b, model group (irradiated rats without drug administration); c, low-dose treatment group (2 g/kg/day); d, intermediate-dose treatment group (6 g/kg/day); e, high-dose treatment group (18 g/kg/day); MMP-12, matrix metalloproteinase-12; TIMP-1, tissue inhibitors of matrix metalloproteinase-1; p.i., post-irradiation.

To investigate the effects of Yangyinqingfei decoction on mRNA and protein expression of MMP-12 and TIMP-1 in rats with radiation-induced lung injury, RT-PCR and western blot analysis were performed, respectively. As shown in Fig. 2A, the level of MMP-12 mRNA in lung tissues increased following irradiation and peaked at two weeks post-irradiation; however, at four weeks post-irradiation the level of mRNA was reduced markedly. By contrast, the level of TIMP-1 mRNA in lung tissues increased following irradiation and was highest four weeks post-irradiation. In comparison with group B, the levels of MMP-12 and TIMP-1 mRNA in groups C-E were reduced. These results indicate that Yangyinqingfei decoction had an inhibitory effect on mRNA expression of MMP-12 and TIMP-1, particularly at the higher dose.

The protein levels of MMP-12 and TIMP-1 were observed in lung tissue using western blotting. As shown in Fig. 2B, MMP-12 and TIMP-1 proteins were positively expressed in each group. The protein expression levels of MMP-12 and TIMP-1 were significantly increased in group B following irradiation compared with those of group A. Compared with group B, the protein expression of MMP-12 and TIMP-1 was effectively downregulated in groups C-E, particularly in group D; however, expression levels remained higher than those in group A. This result further demonstrates the inhibitory effect of Yangyinqingfei decoction on the expression of MMP-12 and TIMP-1.

## Discussion

In the present study it was demonstrated that Yangyinqingfei decoction exerted a protective effect on rats with radiation-induced lung injury. The mechanism underlying this protective effect may involve the downregulation of MMP-12 and TIMP-1 expression.

Pathological changes in radiation-induced lung injury include interstitial pulmonary congestion and edema, inflammatory cell infiltration, interstitial lung fiber hyperplasia and alveolar atrophy (21). At present, radiation-induced lung injury is a well-established model in rats that has been used in numerous studies (22,23). In the present study, Wistar rats were irradiated to induce lung injury. One week after irradiation, alveolar wall edema and capillary congestion were observed. Two weeks post-irradiation, macrophage infiltration was observed. Alveolar septal thickening and alveolar structure disappeared four weeks after irradiation. Lesions were dominated by signs of chronic inflammation, visible fibroblast hyperplasia and alveolar septa thickening, as well as the shrinking or compensatory expansion of alveolar space. Thus, the rat model for radiation-induced lung injury was successfully established.

In this study, the results suggested that early intervention with Yangyinqingfei decoction alleviated pulmonary edema, congestion, inflammation and alveolar septal thickening compared with the model group (group B). This indicates that early application of Yangyinqingfei decoction may be effective for the control and the mitigation of radiation-induced lung injury. The analysis of histopathological changes in the lung showed that rats administered a high dose of Yangyinqingfei decoction exhibited reduced interstitial lung congestion, mild edema and less alveolar structural damage compared with the groups administered lower doses. However, rats treated with a high dose of Yangyinqingfei decoction exhibited side effects, including diarrhea. These results indicate that the dosage of Yangyinqingfei decoction has an impact on the efficacy of the treatment. However, despite the side effects, high-dose Yangyinqingfei decoction showed a greater protective effect against radiation-induced lung injury than the doses used in the other groups.

Radiation causes alveolar injury, leading to alveolar interstitial inflammation, cell proliferation and apoptosis. The pathological repair process in the lung tissue includes interstitial lung cell proliferation, ECM deposition and lung tissue remodeling (24). Excessive deposition of ECM is a key factor leading to pulmonary fibrosis. ECM is degraded by proteases, of which MMPs are the most important and are capable of degrading almost all the components of the ECM. Studies have shown that MMPs are involved in a wide range of biological activities, including cell migration, angiogenesis and atherosclerosis. MMPs have also been reported to be involved in the invasion and metastasis of malignant tumors, as well as other pathological processes, in a variety of fibrotic diseases (25–28). In the present study, the mRNA and protein expression of MMP-12 and TIMP-1 increased in the model group following radiation. This may have been the result of the radiation damage to the endothelial cells, which led to the accumulation of a series of inflammatory cells, including macrophages, neutrophils and lymphocytes, in the alveolar cavity. These cells release a series of cytokines, growth factors and inflammatory mediators to promote the synthesis and activation of MMPs. Wright *et al* (29) reported that MMP-12 expression significantly increased in mice exposed to cigarette smoke; however, in mice lacking tumor necrosis factor-α (TNF-α) receptors, expression of MMP-12 did not change, indicating that TNF-α can activate the expression of MMP-12. Another study indicated that MMP-12 is a pro-inflammatory factor, which is capable of inducing expression of other MMPs, including MMP-2 and MMP-9, and activating the MMP-12 hydrolysis cascade, leading to the degradation of other ECM components and causing the dissolution of the basement membrane in lung tissue (30). Destruction of the integrity of the basement membrane, as well as alveolar and interstitial injuries caused by various factors, leads to acute radiation-induced lung injury (31). In addition, the destruction of the basement membrane, which regulates lung epithelial permeability, may promote migration of fibroblasts, as well as deposition of the alveolar cavity collagen fiber, and cause pulmonary fibrosis. TIMPs are endogenous inhibitors of MMPs, and it has been demonstrated that the MMP/TIMP and ECM metabolic imbalance is involved in the development and progression of pulmonary fibrosis (32). Selman *et al* (33) showed that in patients with IPF, dominant active TIMP expression resulted in filamentous collagen degradation, which caused the occurrence of pulmonary fibrosis. Using a macrophage-dependent immunoglobulin A immune complex mouse model, Gibbs *et al* (34) demonstrated that lung damage in this model may be inhibited by TIMP-2. These studies not only suggest that macrophages may be the source of MMPs in the early stages of lung damage, but also suggest that TIMPs may have a role in the prevention of injury during the early stages of lung damage (35). In the present study, increased expression of MMP-12 and TIMP-1 mRNA was observed in lung tissue during the first four weeks post-irradiation. However, at four weeks post-irradiation, MMP-12 expression was reduced while expression of TIMP-1 remained elevated, which may have led to an MMP-12/TIMP-1 imbalance and the accumulation of ECM fibrosis. Compared with the irradiation group without drug treatment (group B), mRNA and protein expression of MMP-12 and TIMP-1 in the lungs of rats in the Yangyinqingfei decoction intervention groups were lower at each time-point, thus maintaining the MMP-12/TIMP-1 balance. Accordingly, this led to the balance of synthesis and degradation of the ECM, alleviating the occurrence of pulmonary fibrosis.

In conclusion, this study showed that Yangyinqingfei decoction has a preventative and therapeutic effect on the early phase of radiation-induced lung injury, the mechanism of which may involve the suppression of MMP-12 and TIMP-1. However, more detailed studies are required in order to further evaluate the mechanisms underlying the observed effects.

## Figures and Tables

**Table I:** Body weight of rats following irradiation with or without Yangyinqingfei decoction administration.

		Body weight (g)				
		
Time	n	A	B	C	D	E
1 week	5	312.8±1.8	261.2±2.7^a^	273.1±9.3^a^	284.6±9.6^a^,^c^	253.1±16.8^a^
2 weeks	5	347.9±6.6	287.5±5.8^a^	302.7±6.4^a^,^b^	311.3±4.8^a^,^b^	292.8±12.3^a^
4 weeks	5	377.2±7.4	315.0±8.5^a^	333.5±12.3^a^,^c^	344.9±12.3^a^,^b^	324.0±14.3^a^

Data are presented as the mean ± standard deviation.

a P<0.01 compared with group A;

b P<0.01,

c P<0.05 compared with group B. (A) Control group; (B) model group (irradiated rats without drug administration); (C) low-dose treatment group (2 g/kg/day); (D) intermediate-dose treatment group (6 g/kg/day); (E) high-dose treatment group (18 g/kg/day).
